# Associations of maternal dietary carbohydrate intake, glycemic index, and glycemic load during pregnancy with offspring neurodevelopment

**DOI:** 10.1007/s00431-025-06519-5

**Published:** 2025-10-30

**Authors:** Javier Mateu-Fabregat, Josefa Canals, Cristina Jardí, Carmen Hernández-Martínez, Núria Voltas, Mònica Bulló, Victoria Arija

**Affiliations:** 1 Nutrition and Metabolic Health Research Group (NuMeH), Department of Biochemistry and Biotechnology, Universitat Rovira I Virgili (URV), Reus, Spain; 2 Center of Environmental, Food and Toxicological Technology (TecnATox), Universitat Rovira I Virgili (URV), Reus, Spain; 3 Institute of Health Pere Virgili (IISPV), Reus, Spain; 4 Nutrition and Mental Health Research Group (NUTRISAM), Universitat Rovira I Virgili (URV), Reus, Spain; 5 Research Center for Behavior Assessment (CRAMC), Departament of Psychology, Universitat Rovira I Virgili (URV), Tarragona, Spain; 6 Serra Húnter Fellow, Department of Psychology, Universitat Rovira I Virgili (URV), Tarragona, Spain; 7 CIBER Physiology of Obesity and Nutrition (CIBEROBN), Carlos III Health Institute, Madrid, Spain

**Keywords:** Carbohydrates, Glycemic index, Glycemic load, Neurodevelopment, Pregnancy

## Abstract

Emerging evidence suggests that periconceptional nutrition could significantly impact children’s brain development. Few studies have explored the effect of maternal carbohydrate intake on offspring’s cognitive outcomes. Evidence suggests poor glycemic control during pregnancy may impair neurodevelopment. Optimizing maternal carbohydrate intake during pregnancy by focusing on low to moderate glycemic index (GI) and glycemic load (GL) foods may reduce these risks. This study aimed to evaluate the associations between maternal carbohydrate intake, dietary GI, and GL during first and third trimesters of pregnancy and children’s neurodevelopment in the early postnatal period and preschool years. The study included 420 mother-child pairs from the ECLIPSES cohort. Maternal carbohydrate intake, GI, and GL were assessed using a validated food frequency questionnaire during the first and third trimesters. Children’s neurodevelopment was assessed at 40 days of age using the Bayley Scales of Infant Development and at 4 years using the Wechsler Preschool and Primary Scale of Intelligence and the Developmental Neuropsychological Assessment. Multivariable linear and logistic regression analyses were conducted. Maternal carbohydrate intake, GI, and GL were negatively associated with expressive language development in the early postnatal period (*P* < 0.05). Children of mothers in the highest GL tertile during early pregnancy showed lower motor development at 40 days (*β* coefficient [95% CI] = −3.517 [−6.898, −0.135], *P*-trend 0.041). Higher maternal carbohydrate consumption and GL throughout pregnancy were linked to lower processing speed, intelligence quotient, nonverbal skills, and visuomotor precision at preschool age (all *P*-trend < 0.05).

*Conclusion*: The quantity and quality of maternal carbohydrate intake, particularly GL, influence children’s neurodevelopment, including language, motor skills, processing speed, nonverbal abilities, overall intellectual functions, and visuomotor precision, highlighting the need for more refined dietary recommendations during pregnancy.

**Table:** 

**What is known:** • *Maternal nutrition plays a critical role in shaping children'’s brain health*. • *Poor maternal glycemic control has been linked to adverse neurodevelopmental outcomes, but the specific impact of maternal carbohydrate quantity and quality remains unclear*. **What is New:** • *The quantity and quality of maternal carbohydrate intake influence children'’s neurodevelopment in early life*. • *These findings support the need for tailored dietary recommendations during pregnancy focusing on carbohydrate quantity and quality to optimize neurodevelopmental outcomes*.

**Supplementary Information:**

The online version contains supplementary material available at 10.1007/s00431-025-06519-5.

## Introduction

Human brain development involves complex genetic, environmental, and molecular processes, starting as early as the third week of pregnancy and continuing through adolescence and potentially throughout life [1, 2]. A healthy maternal lifestyle is crucial not only for preventing pregnancy complications but also for optimizing central nervous system development in offspring [3, 4]. Among lifestyle factors, prenatal and periconceptional nutrition has garnered significant attention for its impact on children’s brain development and the prevention of neurodevelopmental disorders [1, 5]. Certain maternal dietary patterns and nutrients have been shown to positively influence children’s neurodevelopment. For example, adherence to a Mediterranean diet, rich in vegetables, fruits, olive oil, nuts, and whole grains, has been associated with higher intelligence quotients, improved cognitive and social-emotional outcomes, and a reduced risk of depressive and externalizing problems, such as attention and aggressive behaviours in children [4, 6, 7]. Similarly, adequate nutritional status of certain micronutrients such as vitamin B12, iron, or folate and specific macronutrients like polyunsaturated fatty acids has been linked to improved neurodevelopmental outcomes [8–10]. In contrast, a higher consumption of ultra-processed foods, often rich in saturated fats, additives, salt, and refined starches, has been associated with poorer performance in specific cognitive domains, including verbal function [11]. However, although carbohydrates provide more than half of the total energy intake in the human diet, these studies did not specifically examine the impact of maternal carbohydrate consumption during pregnancy on offspring cognitive outcomes. Beyond the total amount of dietary carbohydrate intake, the quality of carbohydrates, assessed by the glycemic index (GI) and glycemic load (GL), plays a crucial role in influencing cardiometabolic health. While the GI ranks carbohydrate-containing foods according to how rapidly they increase postprandial blood glucose levels, GL provides a more comprehensive evaluation by accounting for both the rate of glucose increase and the actual amount of carbohydrates per serving. These measures provide a physiologically relevant estimate of a food’s impact on glucose and insulin metabolism and are commonly used as a proxy of carbohydrate quality. Over the years, a growing body of evidence has shown that elevated blood glucose levels and poor glycemic control during pregnancy are linked to impaired neurodevelopmental outcomes, including cognitive and behavioural issues in children [12]. For instance, children of mothers with a periconceptional diet characterized by high GL exhibited more behavioural problems including anxiety or inhibition-related behaviours at around 14 months [13]. While dietary markers of carbohydrate quality have been studied in relation to behavioural risks, research on their impact on cognitive development remains limited, primarily focusing on cases of gestational diabetes mellitus (GDM) and hyperglycemia—both conditions that influence insulin and glucose levels—thereby reinforcing the proposed hypothesis. Given this, optimizing maternal carbohydrate intake by prioritizing low to moderate GI and GL foods may help mitigate potential negative effects on offspring neurodevelopment. We hypothesize that the quantity but more importantly the quality of dietary carbohydrates during pregnancy may adversely affect the child’s neurocognitive development. Therefore, this study aimed to evaluate the associations between maternal dietary carbohydrates quantity and quality during early (12th week) and late (36th week) pregnancy and children’s neurodevelopment in the early postnatal period (after 40 days postpartum) to assess the short-term impact of the pregnancy, and at preschool age (around 4 years old) to reflect the sustained or persistent effects of prenatal environmental factors on child development.

## Material and methods

### Study design

This analysis was conducted within the framework of the ECLIPSES study, a multi-centre, controlled, triple-blind, randomized clinical trial carried out in the province of Tarragona, Spain. The trial aimed to evaluate the effectiveness of different doses of iron supplementation during pregnancy on maternal iron status at the end of gestation. The study included pregnant women without anemia, who were recruited from primary health care centres before the 12th week of pregnancy. Exclusion criteria included multiple pregnancies, iron supplementation exceeding 10 mg during the preceding months, major medical conditions, and several pregnancy risk factors such as a history of adverse obstetric outcomes or suspected fetal malformations, among others [14]. Detailed information on the study has been extensively described elsewhere [14, 15]. In summary, participants were visited by midwives and dietitians at 12, 24, and 36 weeks of pregnancy, during which clinical, dietary, lifestyle, anthropometric, and biochemical data were collected. Additionally, the children underwent neuropsychological assessments at 40 days old and again at 4 years of age.

### Dietary assessment

A semi-quantitative 45-item food frequency questionnaire validated in our population was administered to assess maternal dietary intake during early and late pregnancy [16]. Data were analyzed and converted into grams per day for each item, using average portion sizes based on previous studies in this population [17]. Total energy, macronutrient, and micronutrient intake were calculated using the REGAL composition tables [18], complemented by the Mataix Verdú food composition tables [19]. The GI was estimated using the 2021 international tables with glucose as the reference food [20], following a step-based methodology [21]. The dietary GL for each participant was determined by multiplying the GI of each food item by the available carbohydrates per serving and dividing by 100. Dietary GI was calculated by dividing dietary GL by total carbohydrates intake and multiplying the product by 100. Children’s dietary intake at 4 years of age was assessed using the same methodology.

### Assessment of neurodevelopmental outcomes

The Bayley Scales of Infant and Toddler Development, Third Edition (BSID-III) were administered to infants at approximately 40 days of age to evaluate neurodevelopment [22]. The BSID-III consists of three primary domains: cognitive, language (further divided into receptive and expressive communication), and motor scales (comprising fine and gross motor skills). The cognitive scale assesses various aspects of development, including sensorimotor skills, visual attention, exploration, self-regulation, and habituation to the environment. The raw scores from the general scales were standardized to have a mean of 100 and a standard deviation of 15, while the four subscales were standardized to have a mean of 10 with a standard deviation of 3.

A cognitive abilities assessment was conducted on the children at 4 years of age using the Wechsler Preschool and Primary Scale of Intelligence, Fourth Edition (WPPSI-IV) [23] along with selected subtests from the Developmental Neuropsychological Assessment, Second Edition (NEPSY-II) [24]. The administration of the WPPSI-IV yielded the following primary indexes: Verbal Comprehension Index, Fluid Reasoning Index, Working Memory Index, and Processing Speed Index, as well as the Full-Scale Intelligence Quotient (FSIQ) and three secondary indexes: Vocabulary Acquisition Index, Nonverbal Index, and General Ability Index. Each index is standardized to have a mean of 100 and a standard deviation of 15. To complement the WPPSI-IV, the NEPSY-II subtests for verbal fluency and visuomotor precision were used, with scores standardized to have a mean of 10 and a standard deviation of 3. In interpreting these results, higher scores indicate better performance across the evaluated domains.

### Complementary tests

The International Physical Activity Questionnaire (IPAQ-S) was employed to estimate physical activity levels, calculating the metabolic equivalents of tasks (METs) in minutes per week [25]. The State-Trait Anxiety Inventory (STAI) was used to assess state anxiety, defined as a temporary emotional condition [26]. Smoking status was determined using the Fagerström Test, classifying participants as either smokers or non-smokers [27]. Socioeconomic status was assessed based on participants’ education and occupation, and categorized as low, medium, or high, as previously described [15]. The Matrix Reasoning subscale of the Wechsler Adult Intelligence Scale (WAIS-IV) [28] was administered to at least one parent, or the average of both, when possible, to estimate the parental Intelligence Quotient (IQ) approximation. Adherence to the Mediterranean diet was evaluated using the relative Mediterranean diet (rMED) score, which is based on 9 key components. The rMED scores range from 0 to 18, with higher scores indicating greater adherence to the diet [29]. Information on the infant’s type of feeding at 40 days of age, categorized as breastfeeding, mixed or infant formula, was also recorded.

### Statistical analysis

General characteristics of study population are expressed as mean (standard deviation) or median (P25, P75) for normally and non-normally distributed variables, respectively, and numbers with percentages for categorical variables. Normal distribution was checked by using the Anderson-Darling test and visually using a Q-Q plot. Rank-based inverse normal transformation (RNOmni R package v.1.0.1) was applied to GI, GL and carbohydrates intake to approach a normal distribution (mean = 0, SD = 1). We applied the random forest imputation approach (missForest R package v.1.5) to deal with the missing data in covariates (e.g., maternal body mass index, type of feeding, child’s weight, and energy intake) The effectiveness of the imputation was checked visually using density plots comparing observed and imputed data.

Individuals were categorized into tertiles based on their dietary intake of carbohydrates, GI, and GL during early and late pregnancy. Analyses were also conducted using these predictors as continuous variables (per 1-SD increment). To evaluate the relationships between total carbohydrates intake, GI, and GL with neurodevelopment in children at 40 days and 4 years of age, multiple multivariable regression analyses were performed. The model was adjusted for a range of potential confounders identified in prior research, including maternal age, socioeconomic status, intervention group, body mass index at recruitment, state anxiety score (STAI), smoking during pregnancy, gestational weight gain, preterm birth, relative adherence to the Mediterranean diet (rMED), physical activity and energy intake in early or late pregnancy, the child’s weight and sex, Apgar score at 5 min, and type of feeding. For children at 4 years of age, the models were further adjusted for parental IQ approximation, children’s energy intake, and their own GI, GL, or carbohydrates intake, depending on which maternal dietary factor was being assessed. Specifically, models using GI as a predictor were also adjusted for total carbohydrates intake, while models evaluating total dietary carbohydrates intake were adjusted for GI. The results are shown as *β* coefficients along with their 95% confidence intervals (CIs). Interactions between predictors and sex were assessed by including interaction terms in the fully adjusted models. Since none of these interactions were statistically significant (*P* > 0.05), all analyses were performed on the entire study population. To evaluate *P*-trends, the median value of each tertile was assigned and included as a continuous variable in the multivariable models, with the first tertile serving as the reference group. Additionally, multivariable logistic regression analyses (odds ratios [OR] with 95% CIs) were conducted using the FSIQ as a binary outcome, categorizing scores as below 115 or 115 and above. A sensitivity analysis was performed to additionally adjust for maternal intake of fibre, protein, and saturated, monounsaturated, and polyunsaturated fats. Statistical analyses were conducted using R version 4.2.2 (R Foundation for Statistical Computing, Vienna, Austria), with a significance level set at *P* < 0.05.

## Results

**Fig. 1:**
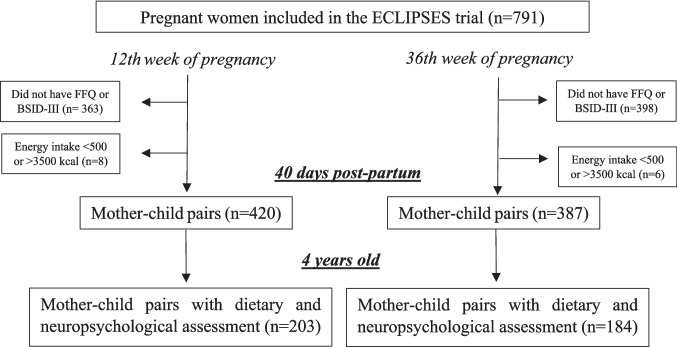
Flowchart of study population. Abbreviations: BSID-III Bayley Scales of Infant and Toddler Development, FFQ food frequency questionnaire (of the mother at 12th or 36th week of pregnancy)

Of the 791 pregnant women included in the ECLIPSES study, 420 mother-offspring pairs with neurodevelopmental and dietary data from the 1st trimester and 387 pairs from the 3rd trimester had total energy intakes within the range of 500 to 3500 kcal per day [30] and were included in the analyses at 40 days of age. For the analysis at 4 years of age, data were available for 203 and 184 pairs, respectively (Fig. 1).

Table 1 shows the maternal characteristics during the first trimester of pregnancy as well as offspring characteristics at birth, 40 days postpartum, and at 4 years of age. The median age of the pregnant women was 31 years. The majority of the women were of normal weight, non-smokers, and classified as having low-to-middle socioeconomic status. Most births were full-term (96.67%), with nearly half of the offspring being female (47.38%). The median birth weight was 3280 g, and a substantial proportion of the children were breastfed.

**Table 1:** Characteristics of the study population

	*N* = 420
***Maternal characteristics (1st trimester of pregnancy)***	
Age (years)	31 [28, 35]
Body mass index (kg/m^2^)	23.73 [21.74, 27.30]
Socioeconomical status, *n* (%)	
Low/middle	337 (80.24)
High	83 (19.76)
Physical activity (METs/min/week)	1386.00 [594.00, 3250.88]
Smoking during pregnancy, *n* (%)	
No	356 (84.76)
Yes	64 (15.24)
Gestational weight gain (kg)	10.20 [8.26, 12.30]
State anxiety score (STAI)	15.25 [11.75, 20.00]
Total energy intake (kcal/day)	1968.73 [1690.37, 2389.39]
Glycemic index	62.13 [53.92, 69.80]
Glycemic load	100.50 [78.38, 125.95]
Carbohydrates intake (g/day)	166.68 [124.78, 205.20]
Relative adherence to the Mediterranean diet score (rMED)	10 [8, 12]
***Children characteristics***	
Weight at birth (g)	3280.00 [3033.75, 3592.50]
Type of feeding, *n* (%)	
Breastfeeding	268 (63.80)
Mixed feeding	55 (13.10)
Infant formula	97 (23.10)
Sex, *n* (%)	
Male	221 (52.62)
Female	199 (47.38)
Apgar 5 min < 7, *n* (%)	1 (0.24)
Preterm birth, *n* (%)	
Yes	14 (3.33)
No	406 (96.67)
*Bayley Scales of Infant and Toddler Development (BSID-III) at 40 days of age*	
Cognitive development score	101.78 (8.8)
Language development score	96.19 (8.34)
Expressive language score	8.04 (1.53)
Receptive language score	10.62 (2.14)
Motor development score	107.46 (11.53)
Fine motor score	11.48 (1.92)
Gross motor score	11.04 (2.37)
*Wechsler Preschool and Primary Scale of Intelligence (WPPSI-IV) at 4 years of age (n* = *203)*	
Verbal Comprehension Index	105.73 (13.17)
Fluid Reasoning Index	102.60 (12.54)
Working Memory Index	98.45 (11.81)
Processing Speed Index	95.59 (12.81)
Vocabulary Acquisition Index	98.04 (13.94)
Nonverbal Index	101.09 (12.01)
General Ability Index	106.33 (11.89)
Full-Scale Intelligence Quotient	102.60 (11.50)
*Developmental Neuropsychological Assessment (NEPSY-II) at 4 years of age (n* = *203)*	
Verbal fluency	9.10 (2.80)
Visuomotor precision	10.16 (3.17)

a Data are expressed as mean (SD) or median (P25, P75) for continuous variables and numbers with percentages for categorical variables

### Associations between maternal dietary carbohydrate intake, GI, GL, and neurodevelopmental outcomes at 40 days of age

Maternal total dietary carbohydrate intake during both early and late pregnancy was significantly associated with reduced expressive language skills in the offspring. Specifically, for each 1-SD increment in carbohydrate consumption, the *β* coefficients [95% CI] were −0.273 [−0.491, −0.055] for early pregnancy and −0.293 [−0.517, −0.070] for late pregnancy (Supplementary Table 1). Additionally, children born to mothers in the highest tertile of carbohydrate intake during late pregnancy had notably lower expressive language scores.

Similarly, increased maternal dietary GI during late pregnancy was associated with lower expressive language skills in the offspring (*β* coefficient [95% CI] = −0.195 [−0.373, −0.017], *P*-value = 0.031). This association was also evident when comparing the medium and highest tertiles to the lowest tertile (*β* coefficient [95% CI] = −0.451 [−0.858, −0.043] and −0.443 [−0.867, −0.019], *P*-trend = 0.051, respectively) (Supplementary Table 2).

Associations of maternal dietary GL in early and late pregnancy with children neurodevelopment at 40 days are shown in Table 2. Children in the highest tertile of maternal GL during early pregnancy showed significantly lower motor development compared to those in the lowest tertile (*β* coefficient [95% CI] = −3.517 [−6.898, −0.135]). Additionally, higher maternal GL during both early and late pregnancy was consistently associated with lower expressive language scores compared to the first tertile (*β* coefficient [95% CI] = −0.701 [−1.151, −0.252] and −0.611 [−1.083, −0.139], respectively). These associations were also observed per 1-SD increment of GL (Table 2). These results remained significant even after adjusting for additional maternal nutritional factors, including intakes of saturated, monounsaturated and polyunsaturated fatty acids, as well as protein and fiber (Supplementary Table 3).

**Table 2:** Associations of dietary glycemic load during early and late pregnancy with neurodevelopment of children at around 40 days of age

*Bayley Scales (BSID-III)*	Tertiles of glycemic load					
Early pregnancy						
	T1 (*n* = 140)	T2 (*n* = 140)	T3 (*n* = 140)	*P*-trend	*β* per 1-SD increment (95% CI)	*P*-value
Glycemic load (median [P25, P75])	69.33 [61.75, 78.33]	100.50 [93.19, 108.76]	140.27 [125.97, 156.30]			
Cognitive development						
Crude Model 1	Ref Ref	−1.121 (−3.190, 0.947) −0.953 (−3.126, 1.219)	−0.121 (−2.190, 1.947) −0.255 (−2.843, 2.332)	0.973 0.887	0.431 (−0.567, 1.129) 0.455 (−0.699, 1.609)	0.515 0.438
Language development						
Crude Model 1	Ref Ref	−1.128 (−3.090, 0.833) −1.310 (−3.382, 0.763)	−0.828 (−2.790, 1.133) −1.430 (−3.899, 1.039)	0.441 0.273	−0.149 (−0.954, 0.654) −0.361 (−1.464, 0.742)	0.714 0.520
Expressive language						
Crude Model 1	Ref Ref	−0.078 (−0.437, 0.280) −0.205 (−0.582, 0.172)	−0.400 (−0.758, −0.041) −0.701 (−1.151, −0.252)	0.025 0.001	−0.123 (−0.270, 0.023) −0.266 (−0.468, −0.065)	0.100 0.009
Receptive language						
Crude Model 1	Ref Ref	−0.307 (−0.808, 0.194) −0.234 (−0.768, 0.298)	0.121 (−0.379, 0.622) 0.228 (−0.407, 0.863)	0.555 0.428	0.075 (−0.130, 0.281) 0.153 (−0.130, 0.437)	0.471 0.289
Motor development						
Crude Model 1	Ref Ref	−0.428 (−3.144, 2.287) −1.642 (−4.481, 1.196)	1.338 10^–14^ (−2.715, 2.715) −3.517 (−6.898, −0.135)	0.980 0.041	0.437 (−0.673, 1.549) −1.181 (−2.693, 0.330)	0.439 0.125
Fine motor						
Crude Model 1	Ref Ref	−0.278 (−0.728, 0.171) −0.443 (−0.910, 0.022)	0.128 (−0.321, 0.578) 0.359 (−0.915, 0.196)	0.497 0.236	0.125 (−0.059, 0.310) −0.102 (−0.351, 0.146)	0.182 0.420
Gross motor						
Crude Model 1	Ref Ref	0.064 (−0.494, 0.622) −0.179 (−0.766, 0.406)	9.793 10^–15^ (−0.558, 0.558) −0.646 (−1.344, 0.052)	0.985 0.065	0.054 (−0.174, 0.283) −0.252 (−0.564, 0.060)	0.640 0.113
Late pregnancy						
	T1 (*n* = 129)	T2 (*n* = 129)	T3 (*n* = 129)	*P*-trend	*β* per 1-SD increment (95% CI)	*P*-value
Glycemic load (median [P25, P75])	68.63 [60.00, 75.10]	95.24 [89.37, 102.84]	127.08 [117.38, 143.91]			
Cognitive development						
Crude Model 1	Ref Ref	0.232 (−1.933, 2.398) 0.420 (−1.910, 2.752)	0.829 (−1.336, 2.995) 1.074 (−1.631, 3.780)	0.446 0.431	0.179 (−0.708, 1.067) 0.283 (−0.911, 1.479)	0.691 0.640
Language development						
Crude Model 1	Ref Ref	−0.852 (−2.827, 1.121) −0.809 (−2.954, 1.336)	−1.093 (−3.067, 0.8819 −1.180 (−3.670, 1.309)	0.285 0.359	−0.685 (−1.492, 0.121) −0.928 (−2.025, 0.167)	0.095 0.096
Expressive language						
Crude Model 1	Ref Ref	−0.255 (−0.629, 0.117) −0.312 (−0.719, 0.094)	−0.449 (−0.823, −0.0759 −0.611 (−1.083, −0.139)	0.019 0.011	−0.208 (−0.361, −0.055) −0.322 (−0.530, −0.114)	0.007 0.002
Receptive language						
Crude Model 1	Ref Ref	−0.038 (−0.541, 0.463) 0.030 (−0.517, 0.578)	0.062 (−0.440, 0.564) 0.196 (−0.439, 0.832)	0.796 0.532	−0.034 (−0.240, 0.171) −0.005 (−0.286, 0.275)	0.740 0.969
Motor development						
Crude Model 1	Ref Ref	1.581 (−1.255, 4.418) 1.175 (−1.891, 4.241)	2.457 (−0.379, 5.294) 1.947 (−1.612, 5.505)	0.092 0.287	0.560 (−0.604, 1.726) 0.202 (−1.370, 1.776)	0.345 0.800
Fine motor						
Crude Model 1	Ref Ref	−0.131 (−0.612, 0.348) −0.064 (−0.581, 0.453)	0.209 (−0.271, 0.690) 0.320 (−0.280, 0.920)	0.362 0.270	−0.043 (−0.241, 0.153) −0.073 (−0.339, 0.192)	0.663 0.586
Gross motor						
Crude Model 1	Ref Ref	0.465 (−0.112, 1.042) 0.264 (−0.360, 0.889)	0.317 (−0.259, 0.895) −0.072 (−0.797, 0.653)	0.309 0.795	0.140 (−0.096, 0.377) −0.00005 (−0.321, 0.320)	0.246 0.999

^a^Results are presented as *β* coefficients and their 95% confidence intervals (CI). ^b^Model 1 was adjusted for maternal age, socioeconomical status, intervention group, body mass index at recruitment, state anxiety score (STAI), smoking during pregnancy, gestational weight gain, preterm birth, relative adherence to Mediterranean diet (rMED), physical activity and energy intake in early or late pregnancy, child’s weight and sex, Apgar at 5 min, and type of feeding

### Associations between maternal dietary carbohydrate intake, GI, GL, and neurodevelopmental outcomes at 4 years of age

We examined the associations between carbohydrate intake during early and late pregnancy and offspring neurodevelopment outcomes. Higher maternal carbohydrate consumption mostly in late pregnancy was linked to lower scores in processing speed index, FSIQ, nonverbal index, and visuomotor precision in the child (all *P*-trend < 0.05) (Supplementary Table 4). Unexpectedly, also in late pregnancy there was a significant positive association between the highest tertile of GI and the processing speed index (Supplementary Table 5). Children born to mothers in the highest tertile of GL during early pregnancy exhibited significantly lower scores in processing speed index (*β* coefficient [95% CI] = −9.902 [−15.075, −4.729]), nonverbal index (*β* coefficient [95% CI] = −5.587 [−10.791, −0.382]), and FSIQ (*β* coefficient [95% CI] = −5.330 [−10.119, −0.540]) compared to those in the lowest tertile (Table 3). Sensitivity analyses indicated that adjusting for other maternal nutrient intakes did not alter these findings (Supplementary Table 3). Additionally, we found that mothers in the highest tertile of GL during early pregnancy were less likely to have children with a FSIQ score 1-SD above the average (≥ 115) compared to those in the lowest tertile (OR [95% CI] = 0.18 [0.03, 0.99]). This association was only significant in early pregnancy (Supplementary Fig. 1B). Furthermore, children born to mothers in the second tertile of carbohydrate intake during late pregnancy, but not those in the highest tertile, had lower odds of scoring 1-SD above the average in FSIQ (OR [95% CI] = 0.16 [0.03, 0.78]) (Supplementary Fig. 1 C). No significant associations were found for GI (Supplementary Fig. 1 A).

**Table 3:** Associations of dietary glycemic load during pregnancy on neurodevelopment of children at 4 years of age

*Wechsler Preschool and Primary Scale of Intelligence (WPPSI-IV)*	Tertiles of glycemic load					
Early pregnancy						
	T1 (*n* = 68)	T2 (*n* = 67)	T3 (*n* = 68)	*P*-trend	*β* per 1-SD increment (95% CI)	*P*-value
Glycemic load (median [P25, P75])	69.19 [60.51, 78.19]	98.81 [93.40, 109.12]	136.20 [123.35, 161.73]			
Verbal Comprehension Index						
Crude Model 1	Ref Ref	−3.072 (−7.541, 1.397) −1.250 (−5.837, 3.337)	−2.942 (−7.395, 1.510) −2.722 (−8.246, 2.802)	0.214 0.330	−0.380 (−2.224, 1.462) 0.392 (−2.185, 2.970)	0.684 0.764
Fluid Reasoning Index						
Crude Model 1	Ref Ref	−4.902 (−9.115, −0.687) −3.396 (−7.936, 1.144)	−4.132 (−8.330, 0.066) −1.779 (−7.246, 3.689)	0.069 0.527	−0.866 (−2.617, 0.884) 0.699 (−1.859, 3.258)	0.330 0.590
Working Memory Index						
Crude Model 1	Ref Ref	−0.177 (−4.198, 3.844) 0.358 (−3.940, 4.658)	−1.627 (−5.634, 2.378) −0.515 (−5.692, 4.661)	0.410 0.842	0.276 (−1.376, 1.928) 1.815 (−0.581, 4.211)	0.742 0.136
Processing Speed Index						
Crude Model 1	Ref Ref	−1.385 (−5.703, 2.933) −2.602 (−6.898, 1.693)	−4.622 (−8.924, −0.319) −9.902 (−15.075, −4.729)	0.032 < 0.001	−0.797 (−2.585, 0.991) −2.862 (−5.335, −0.390)	0.381 0.023
Vocabulary Acquisition Index						
Crude Model 1	Ref Ref	−1.900 (−6.647, 2.846) 0.181 (−4.552, 4.916)	−1.756 (−6.485, 2.973) 0.624 (−5.076, 6.326)	0.485 0.828	0.037 (−1.913, 1.988) 2.177 (−0.459, 4.812)	0.970 0.105
Nonverbal Index						
Crude Model 1	Ref Ref	−2.317 (−6.357, 1.724) −1.820 (−6.141, 2.501)	−4.695 (−8.721, −0.669) −5.587 (−10.791, −0.382)	0.022 0.034	−0.964 (−2.638, 0.709) −0.933 (−3.383, 1.516)	0.257 0.453
General Ability Index						
Crude Model 1	Ref Ref	−3.878 (−7.890, 0.134) −2.218 (−6.310, 1.874)	−3.427 (−7.424, 0.570) −2.918 (−7.846, 2.010)	0.111 0.244	−0.425 (−2.088, 1.237) 0.470 (−1.833, 2.774)	0.615 0.687
Full-Scale Intelligence Quotient						
Crude Model 1	Ref Ref	−2.556 (−6.435, 1.324) −1.649 (−5.626, 2.327)	−4.153 (−8.018, −0.287) −5.330 (−10.119, −0.540)	0.037 0.028	−0.633 (−2.240, 0.974 −0.542 (−2.801, 1.716)	0.438 0.636
***Developmental Neuropsychological Assessment (NEPSY-II)***						
Verbal fluency						
Crude Model 1	Ref Ref	0.264 (−0.687, 1.215) 0.471 (−0.519, 1.463)	0.718 (−0.229, 1.666) 0.575 (−0.618, 1.768)	0.133 0.343	0.220 (−0.170, 0.612) 0.147 (−0.409, 0.704)	0.267 0.602
Visuomotor precision						
Crude Model 1	Ref Ref	−0.810 (−1.882, 0.260) −0.964 (−2.106, 0.177)	0.157 (−0.910, 1.225) −0.618 (−1.993, 0.756)	0.680 0.380	0.021 (−0.422, 0.465) −0.531 (−1.172, 0.108)	0.924 0.102
Late pregnancy						
	T1 (*n* = 62)	T2 (*n* = 60)	T3 (*n* = 62)	*P*-trend	*β* per 1-SD increment (95% CI)	*P*-value
Glycemic load (median [P25, P75])	73.00 [62.62, 79.80]	98.55 [91.17, 103.97]	123.31 [114.22, 136.89]			
***Wechsler Preschool and Primary Scale of Intelligence (WPPSI-IV)***						
Verbal Comprehension Index						
Crude Model 1	Ref Ref	−1.984 (−6.951, 2.984) −0.246 (−5.300, 4.807)	−1.871 (−6.797, 3.055) −0.544 (−6.171, 5.081)	0.451 0.848	−0.157 (−2.197, 1.882) 0.908 (−1.615, 3.432)	0.879 0.478
Fluid Reasoning Index						
Crude Model 1	Ref Ref	2.237 (−2.402, 6.875) 3.146 (−1.777, 8.068)	2.806 (−1.794, 7.407) 2.957 (−2.524, 8.438)	0.228 0.275	1.361 (−0.537, 3.260) 1.576 (−0.888, 4.040)	0.159 0.208
Working Memory Index						
Crude Model 1	Ref Ref	−1.740 (−6.097, 2.617) −1.347 (−6.047, 3.353)	−0.838 (−5.160, 3.482) 0.559 (−4.674, 5.793)	0.697 0.854	−0.535 (−2.322, 1.251) 0.439 (−1.915, 2.795)	0.555 0.712
Processing Speed Index						
Crude Model 1	Ref Ref	−1.349 (−5.995, 3.297) 0.379 (−4.408, 5.168)	−0.451 (−5.059, 4.156) −1.920 (−7.251, 3.411)	0.843 0.491	−0.363 (−2.268, 1.541) −1.375 (−3.767, 1.017)	0.707 0.258
Vocabulary Acquisition Index						
Crude Model 1	Ref Ref	−0.792 (−5.961, 4.377) 1.856 (−3.323, 7.035)	−1.483 (−6.611, 3.643) 1.260 (−4.507, 7.026)	0.567 0.652	0.153 (−1.966, 2.273) 1.962 (−0.615, 4.538)	0.887 0.134
Nonverbal Index						
Crude Model 1	Ref Ref	−1.267 (−5.656, 3.120) −0.513 (−5.041, 4.015)	0.790 (−3.561, 5.142) −0.196 (−5.238, 4.844)	0.726 0.932	0.455 (−1.345, 2.256) 0.073 (−2.192, 2.338)	0.618 0.949
General Ability Index						
Crude Model 1	Ref Ref	−0.296 (−4.649, 4.101) 0.722 (−3.640, 5.085)	−0.112 (−4.474, 4.248) −0.430 (−5.288, 4.426)	0.958 0.874	0.605 (−1.194, 2.405) 0.910 (−1.268, 3.090)	0.508 0.410
Full-Scale Intelligence Quotient						
Crude Model 1	Ref Ref	−1.928 (−6.118, 2.262) −0.686 (−4.875, 3.503)	−1.435 (−5.591, 2.720) −1.961 (−6.625, 2.702)	0.492 0.409	−0.151 (−1.873, 1.569) 0.001 (−2.062, 2.066)	0.862 0.998
***Developmental Neuropsychological Assessment (NEPSY-II)***						
Verbal fluency						
Crude Model 1	Ref Ref	−0.191 (−1.210, 0.826) 0.100 (−0.973, 1.174)	0.014 (−0.995, 1.024) 0.148 (−1.047, 1.343)	0.980 0.804	−0.017 (−0.434, 0.400) −0.065 (−0.602, 0.471)	0.936 0.810
Visuomotor precision						
Crude Model 1	Ref Ref	−0.939 (−2.100, 0.222) −1.130 (−2.393, 0.132)	−0.994 (−2.146, 0.156) −1.537 (−2.943, −0.130)	0.088 0.030	−0.230 (−0.710, 0.248) −0.530 (−1.166, 0.106)	0.343 0.101

^a^Results are presented as *β* coefficients and their 95% confidence intervals (CI). ^b^Model 1 was adjusted for maternal age, socioeconomical status, intervention group, body mass index at recruitment, state anxiety score (STAI), smoking during pregnancy, gestational weight gain, preterm birth, relative adherence to Mediterranean diet (rMED), physical activity and energy intake in early or late pregnancy, parental intelligence quotient approximation, child’s weight and sex, Apgar at 5 min, type of feeding, energy intake, and glycemic load at 4 years of age

## Discussion

In this sub-study conducted within the ECLIPSES trial, we provide evidence that higher maternal carbohydrate intake and dietary GI and GL during both early and late pregnancy are associated with poorer neurodevelopmental outcomes in newborns at 40 days of life and children during early childhood (at 4 years of age). Specifically, higher maternal intake of carbohydrates as well as carbohydrates with high GI and GL was consistently associated with lower expressive language skills in offspring at 40 days of life. Similarly, higher dietary GL during early pregnancy was associated with reduced motor development in newborns. In children at 4 years, elevated maternal carbohydrate intake and dietary GL were both associated with lower scores in processing speed index, nonverbal index, FSIQ, and visuomotor precision abilities.

Maternal nutrition during pregnancy plays a crucial role in postnatal development, impacting neurodevelopment and cognitive and intellectual skills. Previous studies, including a meta-analysis of observational data, have shown that higher maternal intake of fish, seafood, and fruits, and adherence to a Mediterranean diet during pregnancy are associated with improved child neurodevelopment, particularly in cognitive, visual-spatial, and executive functions [31, 32]. Our findings extend this evidence by showing that higher total carbohydrate intake and increased GL during pregnancy are linked to poorer nonverbal abilities, visuomotor precision abilities, and overall intelligence in infants. Supporting our findings, a Spanish study on 2377 pregnant women linked ultra-processed food intake to lower verbal scores in children aged 4–5 years, with high-GI foods such as sweetened beverages and packaged fruit juices being major contributors [11]. Similarly, in a cohort of 1234 mother-child pairs, higher maternal sucrose and sugar-sweetened beverages intake were associated with reduced nonverbal skills, visual memory, and overall intelligence at a median age of 7.7 years [33].

While the mechanisms linking maternal diet to childhood neurodevelopment remain unclear, our findings highlight the importance of both the quantity and quality of carbohydrates during pregnancy. The roles of GI and GL in glucose and insulin metabolism, inflammation, and oxidation are well-documented [34–36], and may help explain how maternal carbohydrate intake affects offspring neurodevelopment. Maternal metabolic status, such as GDM, is known to impact both metabolic health and neurodevelopment in children [37, 38]. Low-GI and -GL diets have been suggested to prevent GDM and mitigate its adverse effects on neurodevelopment [39]. GDM and elevated fasting glucose have been linked to developmental delays in communication and personal-social domains by age 1, potentially mediated by cord blood C-peptide [40], as well as increased risk of speech/language disorders, developmental coordination issues, and other neurodevelopmental disorders, particularly in non-Hispanic white populations [41]. A meta-analysis further emphasizes the importance of glucose and weight management during pregnancy, as they linked to reduced language abilities, noting that maternal diabetes is associated with reduced expressive and receptive language skills, but not with general language abilities or verbal intelligence [42]. In line with this, our study found that higher maternal dietary GI and GL were negatively associated with newborn expressive language, and at age 4, were more consistently linked to nonverbal skills, visuomotor abilities, and overall intelligence. This may be because the expressive language measured by the Bayley scales captures more basic abilities, such as the child’s predisposition to communicate and initiate social interaction [22]. Glucose alterations during pregnancy have also been associated with poorer psychomotor development in children [12]. For example, maternal hyperglycemia was associated with fine motor skills in a study of 308 pre-schoolers [43], and GDM was associated with lower visual motor scores in the Wide Range Assessment of Visual Motor Abilities (WRAVMA) test among 1151 mother-child pairs [44]. These findings align with our results, showing an inverse relationship between GL and early motor development, as well as reduced processing speed and visuomotor precision at 4 years. Additionally, prenatal exposure to diabetes has been associated with lower FSIQ scores in early and mid-childhood [12]. Similarly, we found that higher maternal GL was associated with lower FSIQ scores and a reduced likelihood of children scoring 1-SD above average at age 4. While a 6-point FSIQ change may be clinically relevant [45], these findings should be interpreted with caution regarding their long-term functional impact. The present study adds to existing evidence by showing that higher maternal carbohydrate intake, particularly from high-GI and high-GL foods, during both early and late pregnancy, is associated with less favourable development in certain cognitive domains. Importantly, our findings suggest that GL, rather than GI, more accurately reflects the glycemic impact and physiological response of a carbohydrate-rich meal. Despite distinct developmental stages in early pregnancy (e.g., neural tube formation and structural development) and in the third trimester (e.g., rapid brain growth and increased influence of external stimuli on neural networks) [2], our results suggest that both the amount and quality of carbohydrates, especially as reflected by GL, influence neurodevelopment throughout pregnancy. While previous postnatal and early childhood studies underscore the short- to medium-term effects of prenatal diet, our findings underscore the need to consider maternal carbohydrate quality throughout pregnancy. Given that the long-term cognitive effects of prenatal nutrition may diminish over time [44], extended follow-up is essential to better understand the developmental trajectory shaped by maternal diet.

Our findings should be interpreted within the context of several limitations. To our knowledge, this is the first study to explore how maternal carbohydrate intake, GI, and GL during early and late pregnancy are related to neurodevelopment in newborns and young children, highlighting both short-term and sustained effects of prenatal diet. However, the observational design of the study limits our ability to establish causality. Second, although we used a validated FFQ to assess maternal dietary intake at two points during pregnancy, the dynamic nature of dietary habits during this period means that pre-pregnancy nutrition, as well as potential reporting or measurement errors, cannot be entirely ruled out. Third, although the study’s multi-centre and community-level design enhances its scope, the specific characteristics of our population, such as socioeconomic status, geographical location, and access to medical support during pregnancy, may affect the generalizability of our findings to other societies, countries, and populations with differing dietary patterns, especially those with higher consumption of high-GI foods.

## Conclusion

The study findings reveal that not only the amount but also the quality of carbohydrates consumed during pregnancy, particularly as measured by GL, is linked to neurodevelopment in newborns and young children. These insights emphasize the need to develop and implement community-based nutritional recommendations and policies specifically targeting carbohydrate intake during pregnancy. By promoting balanced carbohydrate consumption and emphasizing high-quality carbohydrate sources, community programs can support improved cognitive outcomes and overall development in children. However, further research through observational studies and specifically designed randomized controlled trials is essential to validate these findings and to refine dietary recommendations, including those issued by the Spanish Agency for Food Safety and Nutrition (AESAN), [46] as well as guidance from other international authorities. Adopting these evidence-based strategies at the community level can lead to significant long-term health benefits for future generations.

## Supplementary Information

Below is the link to the electronic supplementary material.

ESM 1 (DOCX 520 KB)

## Data Availability

Data will be available from the corresponding author upon reasonable request.
