# Supplementary material for: Associations of maternal dietary carbohydrate intake, glycemic index, and glycemic load during pregnancy with offspring neurodevelopment
Source: Eur J Pediatr. 2025 Oct 30;184(11):721. doi: 10.1007/s00431-025-06519-5 (PMC12575505; doi:10.1007/s00431-025-06519-5)
Supplement: Supplementary file 1 — (DOCX 520 KB) [file 431_2025_6519_MOESM1_ESM.docx]

**Supplementary Table 1.** Associations of carbohydrates intake during early and late pregnancy on neurodevelopment of children at 40 days of age.

| ***Bayley Scales (BSID-III)*** | Tertiles of carbohydrates intake | | | P-trend | β per 1-SD increment (95% CI) | P-value |
| --- | --- | --- | --- | --- | --- | --- |
|  | T1 (n=140) | T2 (n=140) | T3 (n=140) |  |  |  |
| **Early pregnancy** | | | | | | |
| **Carbohydrates intake (median [P25, P75])** | 111.74 [99.70, 124.70] | 166.68 [151.67, 178.53] | 229.54 [205.40, 256.34] |  |  |  |
| **Cognitive development** |  |  |  |  |  |  |
| Crude  Model 1 | Ref.  Ref. | 1.950 (-0.114, 4.014)  2.240 (0.029, 4.449) | 0.985 (-1.078, 3.049)  1.122 (-1.633, 3.877) | 0.383  0.439 | 0.535 (-0.311, 1.383)  0.683 (-0.563, 1.929) | 0.215  0.281 |
| **Language development** |  |  |  |  |  |  |
| Crude  Model 1 | Ref.  Ref. | -0.935 (-2.898, 1.027)  -1.252 (-3.375, 0.871) | -0.271 (-2.234, 1.691)  -0.862 (-3.510, 1.785) | 0.814  0.530 | -0.047 (-0.852, 0.757)  -0.445 (-1.640, 0.748) | 0.907  0.463 |
| Expressive language |  |  |  |  |  |  |
| Crude  Model 1 | Ref.  Ref. | -0.050 (-0.410, 0.310)  -0.179 (-0.568, 0.210) | -0.192 (-0.553, 0.167)  -0.414 (-0.900, 0.071) | 0.288  0.093 | -0.094 (-0.242, 0.052)  -0.273 (-0.491, -0.055) | 0.207  0.014 |
| Receptive language |  |  |  |  |  |  |
| Crude  Model 1 | Ref.  Ref. | -0.250 (-0.751, 0.251)  -0.220 (-0.767, 0.325) | 0.107 (-0.394, 0.609)  0.138 (-0.543, 0.819) | 0.637  0.678 | 0.083 (-0.122, 0.289)  0.133 (-0.173, 0.441) | 0.426  0.393 |
| **Motor development** |  |  |  |  |  |  |
| Crude  Model 1 | Ref.  Ref. | -0.428 (-3.141, 2.284)  -1.695 (-4.607, 1.216) | 0.835 (-1.877, 3.549)  -2.523 (-6.154, 1.107) | 0.527  0.173 | 0.672 (-0.438, 1.783)  -0.993 (-2.631, 0.643) | 0.235  0.233 |
| Fine motor |  |  |  |  |  |  |
| Crude  Model 1 | Ref.  Ref. | -0.128 (-0.579, 0.3229  -0.288 (-0.766, 0.190) | 0.085 (-0.365, 0.536)  -0.364 (-0.961, 0.231) | 0.684  0.232 | 0.125 (-0.059, 0.310)  -0.084 (-0.353, 0.185) | 0.182  0.538 |
| Gross motor |  |  |  |  |  |  |
| Crude  Model 1 | Ref.  Ref. | 0.157 (-0.401, 0.715)  -0.130 (-0.731, 0.470) | 0.121 (-0.436, 0.679)  -0.553 (-1.305, 0.194) | 0.680  0.143 | 0.088 (-0.139, 0.317)  -0.247 (-0.585, 0.090) | 0.446  0.150 |
| **Late pregnancy** | | | | | | |
|  | T1 (n=129) | T2 (n=129) | T3 (n=129) | P-trend | β per 1-SD increment (95% CI) | P-value |
| **Carbohydrates intake (median [P25, P75])** | 109.09 [95.79, 120.87] | 155.89 [146.12, 163.78] | 210.41 [191.91, 233.68] |  |  |  |
| **Cognitive development** |  |  |  |  |  |  |
| Crude  Model 1 | Ref.  Ref. | -0.217 (-2.378, 1.944)  -0.147 (-2.531, 2.236) | 1.279 (-0.882, 3.440)  1.352 (-1.593, 4.296) | 0.230  0.350 | 0.356 (-0.530, 1.244)  0.358 (-0.925, 1.642) | 0.430  0.583 |
| **Language development** |  |  |  |  |  |  |
| Crude  Model 1 | Ref.  Ref. | -1.953 (-3.921, 0.014)  -2.034 (-4.221, 0.152) | -0.829 (-2.797, 1.138)  -1.254 (-3.955, 1.447) | 0.455  0.396 | -0.599 (-1.407, 0.208)  -1.027 (-2.204, 0.149) | 0.146  0.087 |
| Expressive language |  |  |  |  |  |  |
| Crude  Model 1 | Ref.  Ref. | -0.503 (-0.876, -0.130)  -0.581 (-0.996, -0.167) | -0.317 (-0.690, 0.055)  -0.531 (-1.043, -0.019) | 0.116  0.051 | -0.160 (-0.313, -0.007)  -0.293 (-0.517, -0.070) | 0.040  0.010 |
| Receptive language |  |  |  |  |  |  |
| Crude  Model 1 | Ref.  Ref. | -0.162 (-0.664, 0.339)  -0.111 (-0.672, 0.449) | 0.023 (-0.478, 0.525)  0.097 (-0.595, 0.791) | 0.900  0.761 | -0.053 (-0.259, 0.152)  -0.067 (-0.369, 0.233) | 0.608  0.658 |
| **Motor development** |  |  |  |  |  |  |
| Crude  Model 1 | Ref.  Ref. | 0.829 (-2.011, 3.670)  -0.002 (-3.146, 3.141) | 2.000 (-0.840, 4.840)  0.714 (-3.168, 4.598) | 0.165  0.709 | 0.640 (-0.525, 1.805)  0.124 (-1.567, 1.815) | 0.281  0.885 |
| Fine motor |  |  |  |  |  |  |
| Crude  Model 1 | Ref.  Ref. | 0.054 (-0.427, 0.536)  0.024 (-0.506, 0.554) | 0.116 (-0.365, 0.598)  0.048 (-0.606, 0.704) | 0.635  0.883 | -0.002 (-0.200, 0.194)  -0.042 (-0.327, 0.242) | 0.977  0.769 |
| Gross motor |  |  |  |  |  |  |
| Crude  Model 1 | Ref.  Ref. | 1.772 10^-15^ (-0.578, 0.578)  -0.281 (-0.921, 0.358) | 0.294 (-0.283, 0.873)  -0.145 (-0.936, 0.645) | 0.305  0.741 | 0.118 (-0.118, 0.356)  -0.054 (-0.398, 0.290) | 0.325  0.758 |

^a^Results are presented as β coefficients and their 95% confidence intervals (CI). ^b^Model 1 was adjusted for maternal age, socioeconomical status, intervention group, body mass index at recruitment, state anxiety score (STAI), smoking during pregnancy, gestational weight gain, preterm birth, relative adherence to Mediterranean diet (rMED), physical activity, energy intake and glycemic index in early or late pregnancy, child’s weight and sex, Apgar at 5 min and type of feeding.

**Supplementary Table 2.** Associations of dietary glycemic index during early and late pregnancy on neurodevelopment of children at 40 days of age.

| ***Bayley Scales (BSID-III)*** | Tertiles of glycemic index | | | P-trend | β per 1-SD increment (95% CI) | P-value |
| --- | --- | --- | --- | --- | --- | --- |
|  | T1 (n=140) | T2 (n=140) | T3 (n=140) |  |  |  |
| **Early pregnancy** | | | | | | |
| **Glycemic index (median [P25, P75])** | 51.14 [47.37, 53.88] | 62.13 [60.17, 64.22] | 72.83 [69.90, 76.68] |  |  |  |
| **Cognitive development** |  |  |  |  |  |  |
| Crude  Model 1 | Ref.  Ref. | 0.964 (-1.091, 3.020)  1.128 (-1.023, 3.278) | -1.735 (-3.791, 0.320)  -1.232 (-3.556, 1.091) | 0.102  0.311 | -0.671 (-1.518, 0.174)  -0.545 (-1.520, 0.428) | 0.119  0.271 |
| **Language development** |  |  |  |  |  |  |
| Crude  Model 1 | Ref.  Ref. | -0.700 (-2.660, 1.260)  -1.022 (-3.089, 1.044) | -1.342 (-3.303, 0.617)  -1.045 (-3.278, 1.188) | 0.178  0.353 | -0.239 (-1.044, 0.565)  -0.071 (-1.005, 0.862) | 0.559  0.880 |
| Expressive language |  |  |  |  |  |  |
| Crude  Model 1 | Ref.  Ref. | 0.064 (-0.296, 0.424)  -0.079 (-0.457, 0.298) | -0.157 (-0.517, 0.203)  -0.289 (-0.697, 0.119) | 0.395  0.165 | -0.066 (-0.213, 0.081)  -0.129 (-0.300, 0.040) | 0.380  0.135 |
| Receptive language |  |  |  |  |  |  |
| Crude  Model 1 | Ref.  Ref. | -0.300 (-0.801, 0.201)  -0.270 (-0.802, 0.261) | -0.314 (-0.816, 0.187)  -0.075 (-0.650, 0.499) | 0.217  0.784 | -0.020 (-0.227, 0.185)  0.100 (-0.139, 0.340) | 0.842  0.409 |
| **Motor development** |  |  |  |  |  |  |
| Crude  Model 1 | Ref.  Ref. | 0.785 (-1.924, 3.496)  0.327 (-2.504, 3.160) | -1.042 (-3.753, 1.667)  -1.695 (-4.755, 1.365) | 0.455  0.282 | -0.341 (-1.453, 0.770)  -0.697 (-1.976, 0.582) | 0.546  0.284 |
| Fine motor |  |  |  |  |  |  |
| Crude  Model 1 | Ref.  Ref. | 0.035 (-0.415, 0.487)  -0.087 (-0.554, 0.378) | -0.078 (-0.530, 0.372)  -0.261 (-0.765, 0.242) | 0.734  0.309 | -0.001 (-0.186, 0.183)  -0.093 (-0.304, 0.116) | 0.990  0.381 |
| Gross motor |  |  |  |  |  |  |
| Crude  Model 1 | Ref.  Ref. | 0.235 (-0.322, 0.793)  0.240 (-0.343, 0.824) | -0.021 (-0.579, 0.536)  -0.086 (-0.717, 0.544) | 0.946  0.799 | -0.028 (-0.257, 0.200)  -0.060 (-0.324, 0.202) | 0.807  0.649 |
| **Late pregnancy** | | | | | | |
|  | T1 (n=129) | T2 (n=129) | T3 (n=129) | P-trend | β per 1-SD increment (95% CI) | P-value |
| **Glycemic index (median [P25, P75])** | 52.78 [48.42, 55.17] | 61.64 [59.56, 63.84] | 71.15 [67.96, 76.08] |  |  |  |
| **Cognitive development** |  |  |  |  |  |  |
| Crude  Model 1 | Ref.  Ref. | -1.356 (-3.520, 0.807)  -0.852 (-3.194, 1.489) | 0.604 (-2.768, 1.558)  0.0212 (-2.418, 2.461) | 0.599  0.930 | -0.418 (-1.305, 0.468)  -0.083 (-1.105, 0.939) | 0.355  0.873 |
| **Language development** |  |  |  |  |  |  |
| Crude  Model 1 | Ref.  Ref. | -1.178 (-3.152, 0.795)  -1.670 (-3.818, 0.478) | -0.441 (-2.415, 1.532)  -1.074 (-3.312, 1.164) | 0.677  0.399 | -0.373 (-1.182, 0.436)  -0.581 (-1.519, 0.356) | 0.365  0.223 |
| Expressive language |  |  |  |  |  |  |
| Crude  Model 1 | Ref.  Ref. | -0.279 (-0.654, 0.096)  -0.451 (-0.858, -0.043) | -0.217 (-0.592, 0.158)  -0.443 (-0.867, -0.019) | 0.265  0.051 | -0.105 (-0.259, 0.048)  -0.195 (-0.373, -0.017) | 0.177  0.031 |
| Receptive language |  |  |  |  |  |  |
| Crude  Model 1 | Ref.  Ref. | -0.108 (-0.610, 0.393)  -0.112 (-0.663, 0.438) | 0.085 (-0.416, 0.587)  0.087 (-0.486, 0.661) | 0.728  0.724 | -0.019 (-0.225, 0.186)  -0.006 (-0.246, 0.233) | 0.852  0.958 |
| **Motor development** |  |  |  |  |  |  |
| Crude  Model 1 | Ref.  Ref. | -1.379 (-4.224, 1.464)  -1.171 (-4.257, 1.914) | -0.395 (-3.239, 2.448)  -0.188 (-3.403, 3.025) | 0.800  0.960 | -0.247 (-1.414, 0.918)  0.024 (-1.321, 1.371) | 0.677  0.971 |
| Fine motor |  |  |  |  |  |  |
| Crude  Model 1 | Ref.  Ref. | -0.224 (-0.706, 0.256)  -0.236 (-0.757, 0.285) | -0.046 (-0.527, 0.434)  -0.090 (-0.633, 0.452) | 0.865  0.796 | -0.102 (-0.300, 0.094)  -0.118 (-0.345, 0.109) | 0.306  0.307 |
| Gross motor |  |  |  |  |  |  |
| Crude  Model 1 | Ref.  Ref. | -0.364 (-0.942, 0.213)  -0.357 (-0.985, 0.270) | 0.031 (-0.546, 0.608)  0.023 (-0.631, 0.677) | 0.892  0.856 | 0.051 (-0.186, 0.288)  0.093 (-0.181, 0.367) | 0.672  0.504 |

^a^Results are presented as β coefficients and their 95% confidence intervals (CI). ^b^Model 1 was adjusted for maternal age, socioeconomical status, intervention group, body mass index at recruitment, state anxiety score (STAI), smoking during pregnancy, gestational weight gain, preterm birth, relative adherence to Mediterranean diet (rMED), physical activity, energy intake and carbohydrates intake in early or late pregnancy, child’s weight and sex, Apgar at 5 min and type of feeding.

**Supplamentary Table 3**. Sensitivity analysis adjusting for maternal nutritional factors. Associations of maternal glycemic load during early pregnancy and neurodevelopment at 40 days (BSID-III) and 4 years of age (WPPSI-IV).

|  | Tertiles of glycemic load | | | P-trend |
| --- | --- | --- | --- | --- |
|  | T1 (n=140) | T2 (n=140) | T3 (n=140) |  |
| **Glycemic load (median [P25, P75])** | 69.33 [61.75, 78.33] | 100.50 [93.19, 108.76] | 140.27 [125.97, 156.30] |  |
| ***Bayley Scales (BSID-III)*** | | | | |
| Expressive language | Ref. | -0.180 (-0.592, 0.232) | -0.696 (-1.301, -0.090) | 0.023 |
| Motor development | Ref. | -2.664 (-5.783, 0.454) | -6.019 (-10.598, -1.439) | 0.010 |
|  | T1 (n=68) | T2 (n=67) | T3 (n=68) |  |
| **Glycemic load (median [P25, P75])** | 69.19 [60.51, 78.19] | 98.81 [93.40, 109.12] | 136.20 [123.35, 161.73] |  |
| ***Wechsler Preschool and Primary Scale of Intelligence (WPPSI-IV)*** | | | | |
| Processing Speed Index | Ref. | -1.987 (-6.638, 2.663) | -8.976 (-15.487, -2.465) | 0.007 |
| Nonverbal Index | Ref. | -3.049 (-7.738, 1.640) | -8.551 (-15.115, -1.986) | 0.010 |
| Full-Scale Intelligence Quotient | Ref. | -2.595 (-6.867, 1.676) | -8.560 (-14.539, -2.580) | 0.005 |

^a^Results are presented as β coefficients and their 95% confidence intervals (CI). ^b^The models were adjusted for maternal age, socioeconomical status, intervention group, body mass index at recruitment, state anxiety score (STAI), smoking during pregnancy, gestational weight gain, preterm birth, relative adherence to Mediterranean diet (rMED), physical activity and energy intake in early pregnancy, child’s weight and sex, Apgar at 5 min, type of feeding, as well as maternal intake of saturated fatty acids, monounsaturated fatty acids, polyunsaturated fatty acids, fiber and protein. For children at 4 years of age, models were further adjusted for parental intelligence quotient approximation, children’s energy intake and glycemic load at 4 years of age.

**Supplementary Table 4.** Associations of carbohydrate intake during pregnancy on neurodevelopment of children at 4 years of age.

| ***Wechsler Preschool and Primary Scale of Intelligence (WPPSI-IV)*** | Tertiles of carbohydrates intake | | | P-trend | β per 1-SD increment (95% CI) | P-value |
| --- | --- | --- | --- | --- | --- | --- |
|  | T1 (n=68) | T2 (n=67) | T3 (n=68) |  |  |  |
| **Early pregnancy** | | | | | | |
| **Carbohydrates intake (median [P25, P75])** | 111.15 [100.98, 121.92] | 166.56 [150.19, 177.75] | 217.86 [201.64, 247.58] |  |  |  |
| **Verbal Comprehension Index** |  |  |  |  |  |  |
| Crude  Model 1 | Ref.  Ref. | -2.119 (-6.589, 2.350)  -2.158 (-7.050, 2.733) | -3.398 (-7.851, 1.055)  -1.818 (-7.926, 4.290) | 0.132  0.525 | -0.443 (-2.286, 1.399)  0.343 (-2.421, 3.108) | 0.636  0.806 |
| **Fluid Reasoning Index** |  |  |  |  |  |  |
| Crude  Model 1 | Ref.  Ref. | -3.405 (-7.645, 0.836)  -2.000 (-6.787, 2.788) | -3.543 (-7.768, 0.681)  -1.448 (-7.426, 4.530) | 0.095  0.597 | -0.807 (-2.559, 0.943  0.671 (-2.032, 3.376) | 0.364  0.624 |
| **Working Memory Index** |  |  |  |  |  |  |
| Crude  Model 1 | Ref.  Ref. | -0.834 (-4.861, 3.192)  0.349 (-4.164, 4.863) | -0.804 (-4.816, 3.207)  0.596 (-5.039, 6.233) | 0.688  0.832 | 0.095 (-1.556, 1.748)  1.590 (-0.946, 4.126) | 0.909  0.217 |
| **Processing Speed Index** |  |  |  |  |  |  |
| Crude  Model 1 | Ref.  Ref. | 0.791 (-3.536, 5.119)  -1.481 (-6.102, 3.138) | -3.254 (-7.566, 1.057)  -6.805 (-12.574, -1.035) | 0.147  0.025 | -0.928 (-2.716, 0.859)  -3.099 (-5.711, -0.487) | 0.307  0.020 |
| **Vocabulary Acquisition Index** |  |  |  |  |  |  |
| Crude  Model 1 | Ref.  Ref. | 0.290 (-4.465, 5.046)  2.109 (-2.858, 7.075) | -0.314 (-5.052, 4.423)  3.965 (-2.236, 10.167) | 0.899  0.205 | 0.489 (-1.460, 2.439)  2.656 (-0.131, 5.443) | 0.621  0.061 |
| **Nonverbal Index** |  |  |  |  |  |  |
| Crude  Model 1 | Ref.  Ref. | -2.649 (-6.709, 1.411)  -1.973 (-6.560, 2.614) | -3.622 (-7.667, 0.423)  -3.196 (-8.923, 2.532) | 0.076  0.264 | -1.178 (-2.850, 0.492)  -1.271 (-3.861, 1.319) | 0.166  0.334 |
| **General Ability Index** |  |  |  |  |  |  |
| Crude  Model 1 | Ref.  Ref. | -3.534 (-7.544, 0.476)  -3.230 (-7.530, 1.069) | -3.942 (-7.937, 0.053)  -2.852 (-8.221, 2.516) | 0.050  0.261 | -0.699 (-2.359, 0.961)  -0.063 (-2.504, 2.377) | 0.408  0.959 |
| **Full-Scale Intelligence Quotient** |  |  |  |  |  |  |
| Crude  Model 1 | Ref.  Ref. | -3.119 (-6.990, 0.753)  -3.337 (-7.544, 0.870) | -4.432 (-8.289, -0.575)  -4.321 (-9.574, 0.932) | 0.023  0.094 | -1.022 (-2.625, 0.581)  -1.143 (-3.531, 1.245) | 0.210  0.346 |
| ***Developmental Neuropsychological Assessment (NEPSY-II)*** |  |  |  |  |  |  |
| **Verbal fluency** |  |  |  |  |  |  |
| Crude  Model 1 | Ref.  Ref. | 0.774 (-0.175, 1.725)  0.876 (-0.162, 1.914) | 0.522 (-0.424, 1.469)  0.847 (-0.449, 2.143) | 0.267  0.171 | 0.099 (-0.293, 0.491)  -0.042 (-0.632, 0.548) | 0.619  0.887 |
| **Visuomotor precision** |  |  |  |  |  |  |
| Crude  Model 1 | Ref.  Ref. | 0.730 (-0.339, 1.800)  0.266 (-0.907, 1.440) | -0.382 (-1.448, 0.683)  -1.399 (-2.864, 0.067) | 0.511  0.088 | -0.191 (-0.634, 0.251)  -0.981 (-1.641, -0.321) | 0.395  0.003 |
| **Late pregnancy** | | | | | | |
|  | T1 (n=62) | T2 (n=60) | T3 (n=60) | P-trend | β per 1-SD increment (95% CI) | P-value |
| **Carbohydrates intake (median [P25, P75])** | 113.29 [99.02, 127.45] | 156.54 [150.39, 163.87] | 205.95 [189.90, 224.27] |  |  |  |
| ***Wechsler Preschool and Primary Scale of Intelligence (WPPSI-IV)*** |  |  |  |  |  |  |
| **Verbal Comprehension Index** |  |  |  |  |  |  |
| Crude  Model 1 | Ref.  Ref. | 0.585 (-4.358, 5.529)  -0.866 (-6.128, 4.396) | -3.112 (-8.016, 1.790)  -2.648 (-8.594, 3.299) | 0.199  0.374 | -0.552 (-2.591, 1.485)  0.464 (-2.179, 3.108) | 0.593  0.729 |
| **Fluid Reasoning Index** |  |  |  |  |  |  |
| Crude  Model 1 | Ref.  Ref. | 4.235 (-0.380, 8.849)  2.419 (-2.709, 7.548) | 1.065 (-3.512, 5.641)  -1.051 (-6.847, 4.745) | 0.699  0.670 | 1.485 (-0.411, 3.382)  1.215 (-1.371, 3.801) | 0.124  0.354 |
| **Working Memory Index** |  |  |  |  |  |  |
| Crude  Model 1 | Ref.  Ref. | -0.847 (-5.209, 3.514)  0.028 (-4.831, 4.887) | -1.032 (-5.358, 3.293)  -2.360 (-7.851, 3.131) | 0.642  0.380 | 0.046 (-1.741, 1.835)  0.289 (-2.158, 2.738) | 0.959  0.815 |
| **Processing Speed Index** |  |  |  |  |  |  |
| Crude  Model 1 | Ref.  Ref. | -0.099 (-4.686, 4.486)  -1.556 (-6.409, 3.297) | -4.580 (-9.129, -0.032)  -7.912 (-13.396, -2.427) | 0.044  0.004 | -1.118 (-3.017, 0.780)  -2.068 (-4.539, 0.403) | 0.247  0.100 |
| **Vocabulary Acquisition Index** |  |  |  |  |  |  |
| Crude  Model 1 | Ref.  Ref. | 1.158 (-3.966, 6.285)  0.571 (-4.782, 5.926) | -3.371 (-8.456, 1.714)  -0.969 (-7.019, 5.081) | 0.178  0.733 | -0.317 (-2.437, 1.802)  0.466 (-1.670, 2.603) | 0.768  0.666 |
| **Nonverbal Index** |  |  |  |  |  |  |
| Crude  Model 1 | Ref.  Ref. | -0.063 (-4.447, 4.319)  -3.428 (-8.127, 1.271) | -2.193 (-6.540, 2.153)  -6.210 (-11.520, -0.898) | 0.310  0.022 | 0.624 (-1.175, 2.424)  -0.206 (-2.606, 2.194) | 0.495  0.865 |
| **General Ability Index** |  |  |  |  |  |  |
| Crude  Model 1 | Ref.  Ref. | 0.978 (-3.395, 5.352)  -1.817 (-6.325, 5.692) | -2.064 (-6.402, 2.273)  -3.255 (-8.350, 1.841) | 0.329  0.210 | 0.348 (-1.452, 2.150)  1.053 (-0.756, 2.862) | 0.703  0.252 |
| **Full-Scale Intelligence Quotient** |  |  |  |  |  |  |
| Crude  Model 1 | Ref.  Ref. | -0.417 (-4.573, 3.737)  -2.994 (-7.310, 1.322) | -3.806 (-7.927, 0.315)  -5.685 (-10.563, -0.807) | 0.065  0.022 | -0.411 (-2.131, 1.309)  -0.391 (-2.589, 1.807) | 0.638  0.725 |
| ***Developmental Neuropsychological Assessment (NEPSY-II)*** |  |  |  |  |  |  |
| **Verbal fluency** |  |  |  |  |  |  |
| Crude  Model 1 | Ref.  Ref. | -0.053 (-1.068, 0.962)  -0.438 (-1.554, 0.677) | -0.502 (-1.509, 0.505)  -0.660 (-1.922, 0.600) | 0.318  0.307 | -0.165 (-0.582, 0.251)  -0.272 (-0.833, 0.287) | 0.435  0.337 |
| **Visuomotor precision** |  |  |  |  |  |  |
| Crude  Model 1 | Ref.  Ref. | -0.753 (-1.912, 0.404)  -1.193 (-2.496, 0.109) | -1.221 (-2.370, -0.072)  -1.746 (-3.218, -0.274) | 0.037  0.021 | -0.401 (-0.878, 0.075)  -0.653 (-1.310, 0.002) | 0.098  0.051 |

^a^Results are presented as β coefficients and their 95% confidence intervals (CI). ^b^Model 1 was adjusted for maternal age, socioeconomical status, intervention group, body mass index at recruitment, State-trait anxiety inventary score, smoking during pregnancy, gestational weight gain, preterm birth, relative adherence to Mediterranean diet (rMED), physical activity, energy intake and glycemic index in early or late pregnancy, parental intelligence quotient approximation, child’s weight and sex, Apgar at 5 min, type of feeding, energy intake and carbohydrates intake at 4 years of age.

**Supplementary Table 5.** Associations of dietary glycemic index during pregnancy on neurodevelopment of children at 4 years of age.

| ***Wechsler Preschool and Primary Scale of Intelligence (WPPSI-IV)*** | Tertiles of glycemic index | | | P-trend | β per 1-SD increment (95% CI) | P-value |
| --- | --- | --- | --- | --- | --- | --- |
|  | T1 (n=68) | T2 (n=67) | T3 (n=68) |  |  |  |
| **Early pregnancy** | | | | | | |
| **Glycemic index (median [P25, P75])** | 51.71 [48.54, 54.68] | 62.13 [60.46, 64.54] | 72.32 [70.01, 76.68] |  |  |  |
| **Verbal Comprehension Index** |  |  |  |  |  |  |
| Crude  Model 1 | Ref.  Ref. | 2.027 (-2.451, 6.507)  1.784 (-2.984, 6.551) | -0.586 (-5.049, 3.876)  -0.535 (-5.509, 4.439) | 0.802  0.831 | 0.206 (-1.637, 2.050)  0.393 (-1.662, 2.450) | 0.825  0.705 |
| **Fluid Reasoning Index** |  |  |  |  |  |  |
| Crude  Model 1 | Ref.  Ref. | -0.923 (-5.199, 3.352)  -0.480 (-5.173, 4.212) | -0.632 (-4.892, 3.627)  0.210 (-4.685, 5.105) | 0.768  0.932 | -0.232 (-1.986, 1.522)  0.127 (-1.891, 2.146) | 0.794  0.901 |
| **Working Memory Index** |  |  |  |  |  |  |
| Crude  Model 1 | Ref.  Ref. | 2.977 (-1.019, 6.974)  5.365 (1.035, 9.694) | -0.356 (-4.338, 3.625)  1.390 (-3.127, 5.906) | 0.870  0.550 | 0.399 (-1.252, 2.051)  1.103 (-0.786, 2.992) | 0.634  0.251 |
| **Processing Speed Index** |  |  |  |  |  |  |
| Crude  Model 1 | Ref.  Ref. | 3.288 (-1.052, 7.628)  -0.106 (-4.605, 4.391) | 0.401 (-3.922, 4.725)  -2.685 (-7.378, 2.007) | 0.847  0.259 | 0.200 (-1.591, 1.992)  -0.953 (-2.892, 0.986) | 0.826  0.333 |
| **Vocabulary Acquisition Index** |  |  |  |  |  |  |
| Crude  Model 1 | Ref.  Ref. | -3.186 (-7.900, 1.529)  -3.295 (-8.116, 1.525) | -4.309 (-9.006, 0.389)  -2.986 (-8.015, 2.043 | 0.071  0.242 | -1.167 (-3.112, 0.776)  -0.251 (-2.337, 1.834) | 0.238  0.812 |
| **Nonverbal Index** |  |  |  |  |  |  |
| Crude  Model 1 | Ref.  Ref. | 0.978 (-3.111, 5.069)  0.184 (-4.304, 4.673) | -0.200 (-4.275, 3.874)  -1.194 (-5.877, 3.489) | 0.926  0.615 | 0.413 (-1.265, 2.091)  0.176 (-1.757, 2.109) | 0.628  0.858 |
| **General Ability Index** |  |  |  |  |  |  |
| Crude  Model 1 | Ref.  Ref. | 1.981 (-2.064, 6.027)  1.929 (-2.323, 6.181) | 0.786 (-3.244, 4.818)  0.959 (-3.476, 5.396) | 0.696  0.669 | 0.485 (-1.177, 2.147)  0.702 (-1.127, 2.533) | 0.565  0.449 |
| **Full-Scale Intelligence Quotient** |  |  |  |  |  |  |
| Crude  Model 1 | Ref.  Ref. | 2.947 (-0.953, 6.849)  2.173 (-1.971, 6.317) | 0.997 (-2.889, 4.884)  0.018 (-4.305, 4.343) | 0.608  0.993 | 0.737 (-0.869, 2.343)  0.495 (-1.293, 2.284) | 0.367  0.585 |
| ***Developmental Neuropsychological Assessment (NEPSY-II)*** |  |  |  |  |  |  |
| **Verbal fluency** |  |  |  |  |  |  |
| Crude  Model 1 | Ref.  Ref. | 0.557 (-0.396, 1.510)  0.252 (-0.768, 1.273) | 0.393 (-0.556, 1.343)  0.360 (-0.704, 1.425) | 0.411  0.504 | 0.191 (-0.200, 0.582)  0.216 (-0.222, 0.654) | 0.337  0.332 |
| **Visuomotor precision** |  |  |  |  |  |  |
| Crude  Model 1 | Ref.  Ref. | 1.036 (-0.030, 2.103)  0.665 (-0.484, 1.816) | 1.141 (0.078, 2.204)  1.089 (-0.111, 2.288) | 0.034  0.074 | 0.388 (-0.051, 0.829)  0.340 (-0.156, 0.837) | 0.083  0.178 |
| **Late pregnancy** | | | | | | |
|  | T1 (n=62) | T2 (n=60) | T3 (n=62) | P-trend | β per 1-SD increment (95% CI) | P-value |
| **Glycemic index (median [P25, P75])** | 52.79 [48.71, 55.22] | 61.85 [59.90, 64.53] | 71.04 [68.08, 74.97] |  |  |  |
| ***Wechsler Preschool and Primary Scale of Intelligence (WPPSI-IV)*** |  |  |  |  |  |  |
| **Verbal Comprehension Index** |  |  |  |  |  |  |
| Crude  Model 1 | Ref.  Ref. | 3.501 (-1.449, 8.451)  4.403 (-0.913, 9.718) | 2.395 (-2.515, 7.304)  2.414 (-3.118, 7.946) | 0.340  0.443 | 1.346 (-0.684, 3.376)  1.145 (-1.197, 3.488) | 0.192  0.335 |
| **Fluid Reasoning Index** |  |  |  |  |  |  |
| Crude  Model 1 | Ref.  Ref. | 2.013 (-2.636, 6.663)  4.084 (-1.167, 9.335) | 0.581 (-4.030, 5.193)  1.357 (-4.108, 6.822) | 0.806  0.697 | 0.167 (-1.741, 2.076)  0.470 (-1.855, 2.796) | 0.863  0.690 |
| **Working Memory Index** |  |  |  |  |  |  |
| Crude  Model 1 | Ref.  Ref. | -0.228 (-4.592, 4.135)  3.007 (-1.962, 7.976) | -0.578 (-4.906, 3.749)  3.226 (-1.940, 8.392) | 0.792  0.234 | -0.905 (-2.689, 0.877)  0.550 (-1.636, 2.737) | 0.318  0.619 |
| **Processing Speed Index** |  |  |  |  |  |  |
| Crude  Model 1 | Ref.  Ref. | 2.266 (-2.283, 6.815)  2.287 (-2.677, 7.251) | 6.437 (1.924, 10.948)  5.791 (0.629, 10.951) | 0.005  0.026 | 1.794 (-0.093, 3.681)  -8.090 (-20.940, 4.761) | 0.062  0.215 |
| **Vocabulary Acquisition Index** |  |  |  |  |  |  |
| Crude  Model 1 | Ref.  Ref. | 3.844 (-1.276, 8.964)  6.062 (0.660, 11.463) | 4.752 (-0.326, 9.829)  5.546 (-0.069, 11.161) | 0.066  0.065 | 1.482 (-0.626, 3.591)  1.636 (-0.756, 4.028) | 0.167  0.178 |
| **Nonverbal Index** |  |  |  |  |  |  |
| Crude  Model 1 | Ref.  Ref. | 0.553 (-3.842, 4.949)  2.576 (-2.282, 7.433) | 1.134 (-3.225, 5.493)  1.655 (-3.365, 6.706) | 0.607  0.553 | 0.183 (-1.619, 1.985)  0.180 (-1.954, 2.314) | 0.841  0.868 |
| **General Ability Index** |  |  |  |  |  |  |
| Crude  Model 1 | Ref.  Ref. | 3.349 (-1.018, 7.716)  4.495 (-0.122, 9.112) | 2.534 (-1.797, 6.865)  1.897 (-2.903, 6.698) | 0.252  0.511 | 1.159 (-0.634, 2.953)  0.685 (-1.357, 2.729) | 0.204  0.508 |
| **Full-Scale Intelligence Quotient** |  |  |  |  |  |  |
| Crude  Model 1 | Ref.  Ref. | 2.746 (-1.420, 6.911)  3.794 (-0.672, 8.261) | 3.471 (-0.660, 7.603)  3.117 (-1.527, 7.760) | 0.098  0.213 | 1.178 (-0.534, 2.890)  0.657 (-1.314, 2.628) | 0.176  0.511 |
| ***Developmental Neuropsychological Assessment (NEPSY-II)*** |  |  |  |  |  |  |
| **Verbal fluency** |  |  |  |  |  |  |
| Crude  Model 1 | Ref.  Ref. | 0.400 (-0.615, 1.415)  0.177 (-0.950, 1.305) | 0.574 (-0.432, 1.581)  0.213 (-0.959, 1.385) | 0.261  0.725 | 0.324 (-0.090, 0.738)  0.195 (-0.297, 0.688) | 0.125  0.435 |
| **Visuomotor precision** |  |  |  |  |  |  |
| Crude  Model 1 | Ref.  Ref. | 1.663 (0.517, 2.810)  1.114 (-0.217, 2.445) | 1.070 (-0.066, 2.206)  0.425 (-0.959, 1.809) | 0.069  0.617 | 0.431 (-0.045, 0.907  0.129 (-0.458, 0.716) | 0.075  0.665 |

^a^Results are presented as β coefficients and their 95% confidence intervals (CI). ^b^Model 1 was adjusted for maternal age, socioeconomical status, intervention group, body mass index at recruitment, state anxiety score (STAI), smoking during pregnancy, gestational weight gain, preterm birth, relative adherence to Mediterranean diet (rMED), physical activity, energy intake and carbohydrates intake in early or late pregnancy, parental intelligence quotient approximation, child’s weight and sex, Apgar at 5 min, type of feeding, energy intake and glycemic index at 4 years of age.


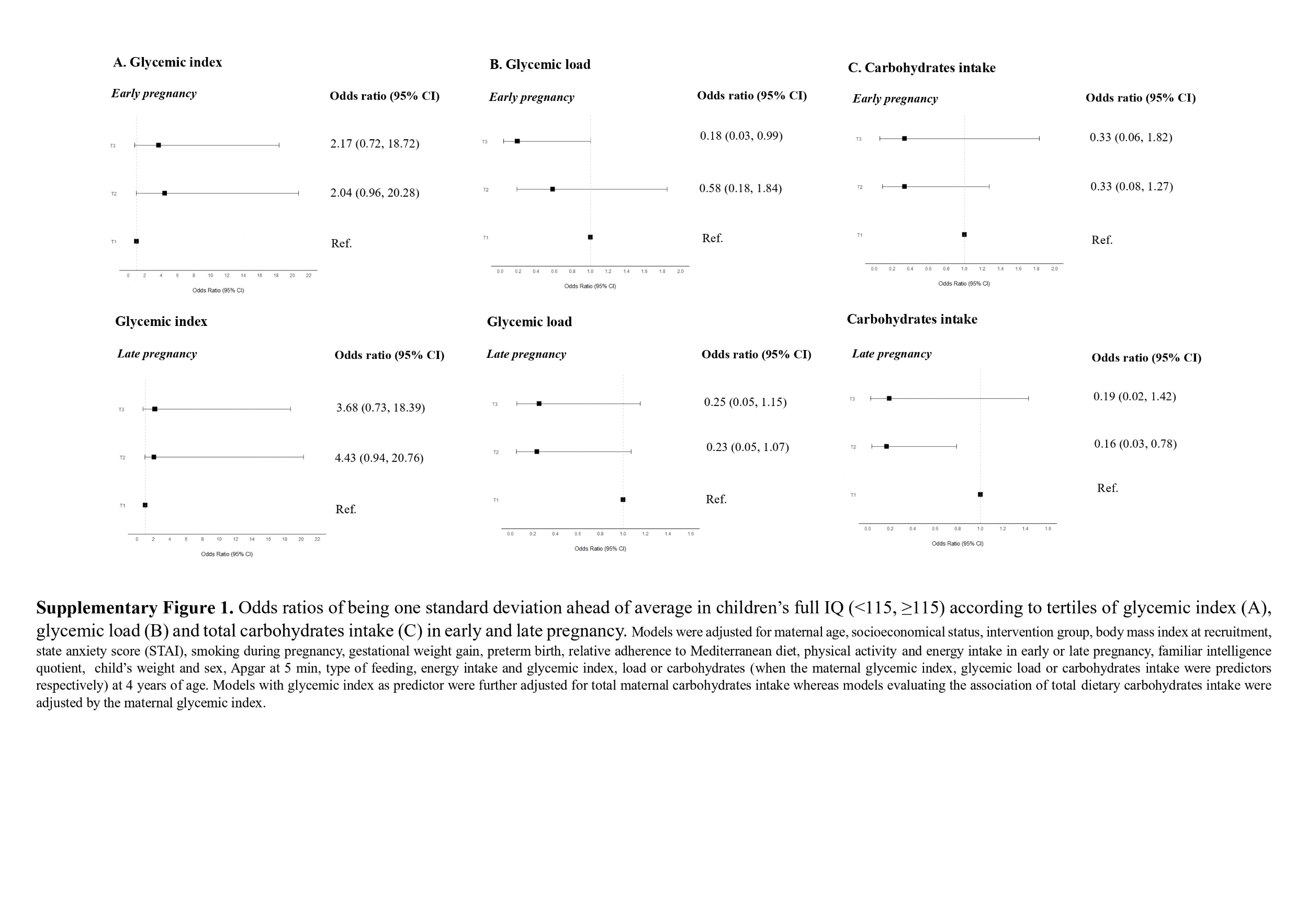
**Supplementary Fig. 1.** Odds ratios of being one standard deviation ahead of average in children’s Full-Scale Intelligence Quotient (FSIQ) (<115, ≥115) according to tertiles of glycemic index (A), glycemic load (B) and total carbohydrates intake (C) in early and late pregnancy. Models were adjusted for maternal age, socioeconomical status, intervention stratum, body mass index at recruitment, state anxiety score (STAI), smoking during pregnancy, gestational weight gain, preterm birth, relative adherence to Mediterranean diet, physical activity and energy intake in early or late pregnancy, parental intelligence quotient approximation, child’s weight and sex, Apgar at 5 min, type of feeding, energy intake and glycemic index, load or carbohydrates (when the maternal glycemic index, glycemic load or carbohydrates intake were predictors respectively) at 4 years of age. Models with glycemic index as predictor were further adjusted for total maternal carbohydrates intake whereas models evaluating the association of total dietary carbohydrates intake were adjusted by the maternal glycemic index.
